# Complete Genome Sequence of the Biocontrol Agent Yeast Rhodotorula kratochvilovae Strain LS11

**DOI:** 10.1128/genomeA.00120-18

**Published:** 2018-03-08

**Authors:** Cecilia Miccoli, Davide Palmieri, Filippo De Curtis, Giuseppe Lima, Giuseppe Ianiri, Raffaello Castoria

**Affiliations:** a Dipartimento Agricoltura, Ambiente e Alimenti, Università degli Studi del Molise, Campobasso, Italy

## Abstract

*Rhodotorula kratochvilovae* strain LS11 is a biocontrol agent (BCA) selected for its antagonistic activity against several plant pathogens both in the field and postharvest. Genome assembly includes 62 contigs for a total of 22.56 Mbp and a G+C content of 66.6%. Genome annotation predicts 7,642 protein-encoding genes.

## GENOME ANNOUNCEMENT

Rhodotorula kratochvilovae (formerly known as Rhodosporidium kratochvilovae and subjected to reclassification [1]) strain LS11 is a biocontrol agent red yeast isolated from olives of a local southern Italian cultivar, Gentile di Larino (2). This strain was selected among many environmental isolates for its high antagonistic activity against the postharvest pathogens Botrytis cinerea and Penicillium expansum (3). Due to toxicological, ethical, and technical concerns related to the use of chemical fungicides, biological and integrated control of plant pathogens both in the field and on stored fruit has been an active field of research over the past two decades (4–6). Our studies revealed that R. kratochvilovae LS11 exerts its antagonistic activity through competition for nutrients and space, a primary mechanism based on resistance to oxidative stress and timely colonization of fruit tissue wounds, the main sites of penetration of fungal pathogens into the host, accompanied by the production of lytic enzymes that degrade pathogen cell walls (3, 7, 8). R. kratochvilovae LS11 is compatible with food grade compounds that enhance its antagonistic activity (9, 10), and it is tolerant to fungicides used in postharvest, thus being suitable for integrated control (11–13). Moreover, R. kratochvilovae LS11 is able to resist and degrade patulin (14), a hazardous mycotoxin produced by P. expansum (15, 16), through two independent pathways that form the breakdown products ascladiol and desoxypatulinic acid (14, 17–21).

Whole-genome sequencing was performed by Macrogen using PacBio sequencing technology, with a starting data set of ∼150,000 reads ranging from 35 bp to 43,052 bp for a total of 837 million sequenced bases. Trimmed reads were subjected to k-mer analysis (22) that revealed high homozygosity and an estimated genome size of 22.56 Mbp. *De novo* genome assembly of PacBio-generated reads was performed using Canu (23), which generated 62 contigs covering 22.10 Mbp (97.96% of the predicted genome size). The largest contig measured 1.7 Mbp, and the *N*_50_ was 704 kbp. G+C content was 66.6%. The high quality of the assembly generated was confirmed using the software QUAST (24), by mapping back the reads to the assembly with the Burrows-Wheeler Aligner (BWA) (25), and with BUSCO (26). Genome annotation was performed using the software Augustus trained with model genes found by BUSCO in the assembly, and this first prediction was used as input for MAKER together with 8,294 proteins of Rhodosporidium toruloides available in GenBank (27–29). The output produced was used to retrain the software Augustus, and the procedure was repeated three times until the best sensitivity and specificity were achieved (0.97 and 0.99, respectively). The final gene prediction generated 7,642 gene models that were searched against the Uniprot (description found for 5,046 genes), KEGG (pathway annotation for 4,475 genes), PFAM (functional domain assigned to 5,319 proteins), and gene ontology (GO) (GO class assigned to 5,234 sequences) databases. The availability of the genome sequence of R. kratochvilovae LS11 coupled with tools for genetic manipulation that we developed (30) represents a crucial step toward the understanding of the molecular mechanisms behind the biocontrol activity of this yeast and its ability to degrade the mycotoxin patulin.

### Accession number(s).

This whole-genome shotgun project has been deposited at DDBJ/ENA/GenBank under the accession number PQDI00000000. The version described in this paper is version PQDI01000000.

## References

[1] Wang QM, Yurkov AM, Göker M, Lumbsch HT, Leavitt SD, Groenewald M, Theelen B, Liu XZ, Boekhout T, Bai FY 2015 Phylogenetic classification of yeasts and related taxa within *Pucciniomycotina*. Stud Mycol 81:149–189. doi:10.1016/j.simyco.2015.12.002. 26951631 PMC4777780

[2] De Curtis F 1998 I lieviti nella lotta biologica contro patogeni fungini degli ortofrutticoli in postraccolta: attività e meccanismi d'azione coinvolti. PhD dissertation, University of Bologna, Bologna, Italy.

[3] Castoria R, De Curtis F, Lima G, De Cicco V 1997 β-1,3-Glucanase activity of two saprophytic yeasts and possible mode of action involved as biocontrol agents against postharvest diseases. Postharvest Biol Technol 12:293–300. doi:10.1016/S0925-5214(97)00061-6.

[4] Janisiewicz WJ, Korsten L 2002 Biological control of postharvest diseases of fruits. Annu Rev Phytopathol 40:411–441. doi:10.1146/annurev.phyto.40.120401.130158. 12147766

[5] Spadaro D, Droby S 2016 Development of biocontrol products for postharvest diseases of fruit: the importance of elucidating the mechanisms of action of yeast antagonists. Trends Food Sci Technol 47:39–49. doi:10.1016/j.tifs.2015.11.003.

[6] Droby S, Wisniewski M, Teixidó N, Spadaro D, Jijakli MH 2016 The science, development, and commercialization of postharvest biocontrol products. Postharvest Biol Technol 122:22–29. doi:10.1016/j.postharvbio.2016.04.006.

[7] Lima G, De Curtis F, Castoria R, De Cicco V 1998 Activity of the yeasts *Cryptococcus laurentii* and *Rhodotorula glutinis* against post-harvest rots on different fruits. Biocontrol Sci Technol 8:257–267. doi:10.1080/09583159830324.

[8] Castoria R, Caputo L, De Curtis F, De Cicco V 2003 Resistance of postharvest biocontrol yeasts to oxidative stress: a possible new mechanism of action. Phytopathology 93:564–572. doi:10.1094/PHYTO.2003.93.5.564. 18942978

[9] Lima G, Spina AM, Castoria R, De Curtis F, De Cicco V 2005 Integration of biocontrol agents and food-grade additives for enhancing protection of stored apples from *Penicillium expansum*. J Food Prot 68:2100–2106. doi:10.4315/0362-028X-68.10.2100. 16245713

[10] De Curtis F, De Cicco V, Lima G 2012 Efficacy of biocontrol yeasts combined with calcium silicate or sulphur for controlling durum wheat powdery mildew and increasing grain yield components. Field Crops Res 134:36–46. doi:10.1016/j.fcr.2012.04.014.

[11] Lima G, De Curtis F, Castoria R, De Cicco V 2003 Integrated control of apple postharvest pathogens and survival of biocontrol yeasts in semi-commercial conditions. Eur J Plant Pathol 109:341–349. doi:10.1023/A:1023595529142.

[12] Lima G, Castoria R, De Curtis F, Raiola A, Ritieni A, De Cicco V 2011 Integrated control of blue mould using new fungicides and biocontrol yeasts lowers levels of fungicide residues and patulin contamination in apples. Postharvest Biol Technol 60:164–172. doi:10.1016/j.postharvbio.2010.12.010.

[13] Lima G, De Curtis F, Piedimonte D, Spina AM, De Cicco V 2006 Integration of biocontrol yeast and thiabendazole protects stored apples from fungicide sensitive and resistant isolates of *Botrytis cinerea*. Postharvest Biol Technol 40:301–307. doi:10.1016/j.postharvbio.2006.01.017.

[14] Castoria R, Morena V, Caputo L, Panfili G, De Curtis F, De Cicco V 2005 Effect of the biocontrol yeast *Rhodotorula glutinis* strain LS11 on patulin accumulation in stored apples. Phytopathology 95:1271–1278. doi:10.1094/PHYTO-95-1271. 18943357

[15] Puel O, Galtier P, Oswald IP 2010 Biosynthesis and toxicological effects of patulin. Toxins 2:613–631. doi:10.3390/toxins2040613. 22069602 PMC3153204

[16] Moake MM, Padilla-Zakour OI, Worobo RW 2005 Comprehensive review of patulin control methods in foods. Comp Rev Food Sci Food Safety 4:8–21. doi:10.1111/j.1541-4337.2005.tb00068.x. 33430570

[17] Castoria R, Wright SAI, Droby S 2008 Biological control of mycotoxigenic fungi in fruits, p 311–333. *In* Barkai-Golan R, Paster N (ed.), Mycotoxins in fruits and vegetables Elsevier, San Diego, CA.

[18] Wright SAI, Ianiri G, De Felice DV, Castoria R 2008 A rapid assay for patulin degradation by the basidiomycetous yeast Rhodotorula glutinis strain LS11, COST Action 924, p 19–29. *In* Novel approaches for the control of postharvest diseases and disorders. Proceedings of the International Congress Lo Scarabeo, Turin, Italy.

[19] Castoria R, Mannina L, Durán-Patrón R, Maffei F, Sobolev AP, De Felice DV, Pinedo-Rivilla C, Ritieni A, Ferracane R, Wright SA 2011 Conversion of the mycotoxin patulin to the less toxic desoxypatulinic acid by the biocontrol yeast *Rhodosporidium kratochvilovae* strain LS11. J Agric Food Chem 59:11571–11578. doi:10.1021/jf203098v. 21928828

[20] Ianiri G, Idnurm A, Wright SA, Durán-Patrón R, Mannina L, Ferracane R, Ritieni A, Castoria R 2013 Searching for genes responsible for patulin degradation in a biocontrol yeast provides insight into the basis for resistance to this mycotoxin. Appl Environ Microbiol 79:3101–3115. doi:10.1128/AEM.03851-12. 23455346 PMC3623128

[21] Ianiri G, Idnurm A, Castoria R 2016 Transcriptomic responses of the basidiomycete yeast *Sporobolomyces* sp. to the mycotoxin patulin. BMC Genomics 17:210. doi:10.1186/s12864-016-2550-4. 26956724 PMC4784387

[22] Mapleson D, Garcia Accinelli G, Kettleborough G, Wright J, Clavijo BJ 2017 KAT: a K-mer analysis toolkit to quality control NGS datasets and genome assemblies. Bioinformatics 33:574–576. doi:10.1093/bioinformatics/btw663. 27797770 PMC5408915

[23] Koren S, Walenz BP, Berlin K, Miller JR, Bergman NH, Phillippy AM 2017 Canu: scalable and accurate long-read assembly via adaptive k-mer weighting and repeat separation. Genome Res 27:722–736. doi:10.1101/gr.215087.116. 28298431 PMC5411767

[24] Gurevich A, Saveliev V, Vyahhi N, Tesler G 2013 QUAST: quality assessment tool for genome assemblies. Bioinformatics 29:1072–1075. doi:10.1093/bioinformatics/btt086. 23422339 PMC3624806

[25] Li H, Durbin R 2010 Fast and accurate long-read alignment with Burrows-Wheeler transform. Bioinformatics 26:589–595. doi:10.1093/bioinformatics/btp698. 20080505 PMC2828108

[26] Simão FA, Waterhouse RM, Ioannidis P, Kriventseva EV, Zdobnov EM 2015 BUSCO: assessing genome assembly and annotation completeness with single-copy orthologs. Bioinformatics 31:3210–3212. doi:10.1093/bioinformatics/btv351. 26059717

[27] Zhu Z, Zhang S, Liu H, Shen H, Lin X, Yang F, Zhou YJ, Jin G, Ye M, Zou H, Zou H, Zhao ZK 2012 A multi-omic map of the lipid-producing yeast *Rhodosporidium toruloides*. Nat Commun 3:1112. doi:10.1038/ncomms2112. 23047670 PMC3493640

[28] Morin N, Calcas X, Devillers H, Durrens P, Sherman DJ, Nicaud J-M, Neuvéglise C 2014 Draft genome sequence of *Rhodosporidium toruloides* CECT1137, an oleaginous yeast of biotechnological interest. Genome Announc 2:e00641-14. doi:10.1128/genomeA.00641-14. 25013136 PMC4110759

[29] Hu J, Ji L 2016 Draft genome sequences of *Rhodosporidium toruloides* strains ATCC 10788 and ATCC 10657 with compatible mating types. Genome Announc 4:e00098-16. doi:10.1128/genomeA.00098-16. 26966203 PMC4786659

[30] Abbott EP, Ianiri G, Castoria R, Idnurm A 2013 Overcoming recalcitrant transformation and gene manipulation in *Pucciniomycotina* yeasts. Appl Microbiol Biotechnol 97:283–295. doi:10.1007/s00253-012-4561-7. 23149757

